# Placebo-controlled randomized clinical trial of fish oil’s impact on fatigue, quality of life, and disease activity in Systemic Lupus Erythematosus

**DOI:** 10.1186/s12937-015-0068-2

**Published:** 2015-08-18

**Authors:** Cristina Arriens, Linda S. Hynan, Robert H. Lerman, David R. Karp, Chandra Mohan

**Affiliations:** 1 Division of Rheumatic Diseases, Department of Internal Medicine, University of Texas Southwestern Medical Center, Dallas, TX USA; 2 Departments of Clinical Sciences (Biostatistics) and Psychiatry, University of Texas Southwestern Medical Center, Dallas, TX USA; 3 Functional Medicine Research Center, Metagenics, Inc, Gig Harbor, WA USA; 4 Department of Biomedical Engineering, University of Houston, Houston, TX USA

## Abstract

**Introduction:**

A recent metabolomic screen of sera from patients with Systemic Lupus Erythematosus (SLE) found reduction of antioxidants and substrates for energy generation. These metabolic alterations may underlie one of the most common features of SLE - fatigue. The metabolomic studies also noted reduced omega-3 fatty acids, which are powerful anti- oxidants. This deficiency may be causally related to oxidative stress, inflammation, disease activity, and fatigue in SLE. Supplementation of omega-3 fatty acids using fish oil in SLE has been shown to reduce oxidative stress in other studies. The objective of this study is to evaluate the effect of fish oil supplementation on clinical measures of fatigue, quality of life, and disease activity as part of a randomized clinical trial.

**Methods:**

Fifty SLE patients recruited in outpatient clinics were randomized 1:1 to fish oil supplementation or olive oil placebo, and blinded to their treatment group. At baseline and after 6 months of treatment, RAND Short Form-36 (RAND SF-36), Fatigue Severity Scale (FSS), SLE Disease Activity Index (SLEDAI), and Physician Global Assessment (PGA) were completed; serum was also collected for soluble mediator analysis.

**Results:**

Thirty-two patients completed the study. PGA improved significantly in the fish oil group compared with the placebo group (*p* = 0.015). The RAND SF-36 Energy/fatigue and Emotional well-being scores demonstrated improvement trends (*p* = 0.092 and 0.070). No clear difference was seen in FSS and SLEDAI (*p* = 0.350 and *p* = 0.417). Erythrocyte sedimentation rate and serum IL-12 were reduced (*p* = 0.008 and *p* = 0.058); while serum IL-13 was increased by fish oil supplementation (*p* = 0.033).

**Conclusions:**

In this randomized, placebo-controlled 6-month trial, SLE patients randomized to fish oil supplementation demonstrated improvement in their PGA, RAND SF-36, and some circulating inflammatory markers.

**Trial registration:**

ClinicalTrials.gov Identifier: NCT02021513 (registered 13 December 2013).

## Introduction

Systemic lupus erythematosus (SLE) is a complex autoimmune disease varying widely in attributes and severity [1, 2]. SLE patients differ from healthy individuals on multiple biological levels including immune cell function, single nucleotide polymorphisms, modified gene expression, as well as metabolic cycle intermediate quantity changes [3]. A comprehensive metabolomic scan revealed a reduction in omega-3 fatty acids in SLE patients compared to healthy controls [4]. Previous studies in SLE noted similar reduction of eicosapentaenoic acid (EPA) and docosahexaenoic acid (DHA), the omega-3 fatty acid components of fish oil [5, 6]. These deficiencies have prompted several fish oil supplement clinical trials in SLE with a variety of outcomes assessed. A recent study evaluated the effect of fish oil on flow-mediated dilation, disease activity, sICAM-1, sVCAM-1, IL-6, and fasting lipid panel, and reported negative findings with the exception of LDL elevation in the fish oil group [7]. However, in another study, SLE patients given fish oil had improved flow-mediated dilation, disease activity, and 8- isoprostanes [8]. A study of EPA alone in six lupus nephritis patients noted significant reduction in urinary 8-isoprostane [9]. An elegant study by Groeger et al. details a possible mechanism for omega-3 fatty acids’ anti-oxidant and anti-inflammatory effects [10]. Disease activity improved in patients receiving fish oil as compared to placebo, however erythrocyte sedimentation rate (ESR) did not improve in a study evaluating fish oil and copper in SLE patients [11]. In another study, lupus nephritis patients treated with fish oil exhibited no significant reduction in SLE disease activity or improvement in renal function, but had reduction of VLDL and triglycerides compared to the olive oil placebo group [12]. Utilizing a unique improvement or deterioration scale, another study noted significant improvement in SLE patients administered fish oil compared to olive oil placebo [13]. Disease activity, immune complexes, and proteinuria were not reduced with 2 different doses of fish oil in lupus nephritis patients, however arachidonic acid, leukotriene B4, VLDL, triglycerides, and whole blood viscosity were reduced [14].

Beyond reinforcing the finding of reduced omega-3 fatty acids in SLE patients, our previously reported metabolomics study also uncovered reduction of markers of energy generation and amplification of markers of oxidative stress and inflammation [4]. These alterations are inferred to correlate with fatigue, a patient-centered outcome not reported in the several prior SLE fish oil supplementation studies. Fatigue impacts 86 % of SLE patients and inconsistently correlates with disease activity [15, 16]. Of relevance, supplementation with omega-3 fatty acids has been associated with improvement in quality of life and depressive symptoms in elderly depression [17]. The possibility that the fatty acid profile impacts fatigue through metabolic processes in SLE prompted a randomized placebo-controlled trial of fish oil in SLE.

## Methods

### Patients and setting

Recruitment for this randomized, single-blind placebo controlled clinical trial occurred in outpatient rheumatology and nephrology clinics in a large urban hospital in Dallas, Texas. Patients were 18–64 years old and had SLE according to the 1997 revised ACR criteria [18]. Key exclusion criteria were allergy to fish or fish oil, current use or use within 2 months of fish oil supplements, warfarin or heparin use, and pregnancy. The Institutional Review Board approved the study protocol and all patients provided written informed consent prior to the study (ClinicalTrials.gov Identifier NCT02021513). A total of 50 patients were randomized, 25 to fish oil and 25 to olive oil placebo. The patients continued receiving medical care from their primary care physicians and specialists who were blinded to treatment group during the trial.

### Study design

A 6 month single center, randomized, single-blind (patient unaware of treatment group), placebo-controlled, parallel-group pilot study of fish oil in SLE was conducted. Patients were randomized using a simple block randomization 1:1 to receive fish oil (6 capsules/day equaling 2.25 g EPA and 2.25 g DHA Metagenics, Inc. Gig Harbor, WA) or visually identical placebo (6 capsules/day purified [refined, not extra-virgin] olive oil Metagenics) in addition to their background therapies, with treatment duration of 6 months. The dose chosen was approximately equivalent to the total EPA and DHA dose used in a prior lupus nephritis study [12], as well as multiple other kidney diseases as outlined in a meta-analysis [19]. All fish oil capsules were encapsulated in the same lot under the Norwegian Medicines Agency (Norway’s equivalent of the Food and Drug Administration) with external testing to verify EPA and DHA levels as well as impurity assessment for safety. Patients were allowed to divide their treatment into one or two doses per day, a daily total of 6 capsules. Basic demographic and clinical information was collected at baseline including: gender, race, ethnicity, age, body mass index (BMI), duration of SLE, number of ACR criteria, hypertension, diabetes, and tobacco use. Global dietary routine was also evaluated using Rate Your Plate (RYP), a dietary recall assessment tool. On this scale, 27–45 indicates poor diet, 46–63 is average with ability to improve eating habits, and 64–81 indicates a healthy diet [20]. Use of the following medications was assessed at baseline and study completion: prednisone, hydroxychloroquine, mycophenolate mofetil, azathioprine, cyclophosphamide, statin (HMG-CoA reductase inhibitor), and Angiotensin-converting enzyme inhibitor or angiotensin receptor blocker (ACEI or ARB).

### Efficacy assessments

Disease activity was assessed at baseline and at 6 months using the Safety of Estrogens in Lupus Erythematosus National Assessment – Systemic Lupus Erythematosus Disease Activity Index (SELENA-SLEDAI) score and Physician Global Assessment (PGA) visual analogue scale [21–23]. For both disease activity measures, higher scores indicate worse disease activity with negative change from baseline (6 months – baseline) indicating improvement. The renal parameters of the SELENA-SLEDAI were assessed in the 24 completer patients with lupus nephritis to determine if additional benefits were apparent in this subgroup. Quality of life was assessed with the RAND 36-Item Health Survey Version 1.0 (RAND SF-36) that results in 8 subscales: Physical functioning, Role functioning/physical, Role functioning/emotional, Energy/fatigue, Emotional well-being, Social functioning, Pain, and General health [24]. Higher scores indicate a better quality of life and therefore a positive change from baseline denotes improvement. In addition to the Energy/fatigue subscale of the RAND SF-36, fatigue was assessed with the Fatigue Severity Scale (FSS) with lower scores indicating less fatigue and negative change from baseline denoting improvement [25]. Both scales have been utilized in prior evaluations of SLE patients [26]. The scores were all assessed by the same evaluator (unblinded) for consistency.

### Biomarker assessments

Baseline and 6 month serum soluble mediator panels were assessed (EGF, eotaxin, FGF-basic, G-CSF, GM-CSF, HGF, IFN-alpha, IFN-gamma, IL-10, IL-12, IL-13, IL-15, IL-17, IL-1beta, IL- 1RA, IL-2, IL-2R, IL-4, IL-5, IL-6, IL-7, IL-8, IP-10/CXCL10, MCP-1/CCL2, MIG/CXCL9, MIP-1alpha/CCL3, MIP-1beta/CCL4, RANTES/CCL5, TNFalpha, and VEGF) using the Luminex magnetic 30-plex human cytokine panel (Luminex ®, Austin, TX). Additionally, serum malondialdehyde (MDA) was assessed using the Thiobarbituric Acid Reactive Substances (TBARS) assay (Cayman Chemical Company, Ann Arbor, MI). Available clinical laboratory data were also evaluated including double-stranded DNA antibody (Anti-dsDNA), complement 3 (C3), complement 4 (C4), creatinine, ESR, C - reactive protein (CRP), lipid panel, and spot urine protein to creatinine ratio.

### Statistical analysis plan

SPSS V21 was used to analyze these data. Sample size was determined using previously reported fish oil studies carried out using different dosage, patient populations, and assessments [8, 9, 11–14, 17]. Based on a target sample size of 20 patients in each group at completion and assuming a 20 % drop out rate, 25 patients were randomized to each arm of the trial. Modified intention-to-treat analyses were performed and included all patients with more than baseline data. Categorical measures were compared using Chi-square or Fisher’s Exact test, as appropriate, and continuous measures using either a *t*-test, Mann–Whitney, ANOVA, or Kruskal-Wallis, as appropriate. Difference scores were calculated for all continuous measures subtracting baseline measures from 6 month measures (end of study-baseline).

## Results

### Patient population

**Fig. 1:**
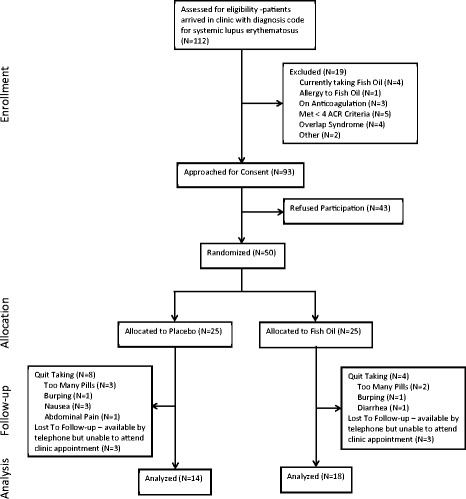
Trial Profile. Flow diagram of progress through phases of the randomized clinical trial

One hundred and twelve patients presenting with a diagnosis code for SLE were assessed for eligibility and their progression through the phases of the study is detailed in the flow diagram (Fig. 1). Nineteen were excluded for the following reasons: currently taking fish oil (*n* = 4), allergy to fish oil (*n* = 1), anticoagulation (*n* = 3), met less than 4 ACR criteria for SLE (*n* = 5), overlap syndrome (*n* = 4), and other (*n* = 2). Ninety-three patients were approached for consent and 43 refused. Fifty patients were recruited and randomized 1:1 to fish oil treatment or olive oil placebo. Randomization resulted in two groups with similar demographics with the exception of higher age, BMI, and hypertension comorbidity in the fish oil group compared to the placebo group (Table 1). Overall, patients were 84 % female, with a median age of 38.79 (range: 19.80–57.65), 44 % Hispanic/Latino, 54 % Black/African American, 2 % White. Median BMI was 29.3 (range: 17.2–49.7), at the high end of being overweight. At baseline the median RYP score was 56.5 (range: 39–75), corresponding with average diet and ability to improve eating habits, with no significant difference between groups. Background medications at baseline and completion did not significantly differ between groups.

**Table 1:** Baseline demographic and clinical features of the placebo and treatment groups

	All		Completers	
	Placebo (*N*=25)	Fish oil (*N*=25)	Placebo (*N*=14)	Fish oil (*N*=18)
Demographics				
Sex, female	22 (88 %)	20 (80 %)	11 (79 %)	14 (78 %)
Age, years	36.1 [28.6–41.0]^a^	46.1 [34.7–49.3]^a^	35.6 [26.3–42.7]^a^	46.2 [36.8–49.1]^a^
Ethnicity				
Hispanic/Latino	11 (44 %)	11 (44 %)	7 (50 %)	8 (44 %)
Black/African American	13 (52 %)	14 (56 %)	6 (43 %)	10 (56 %)
White	1 (4 %)	0 (0 %)	1 (7 %)	0 (0 %)
BMI	25.0 [23.4–32.6]^a^	31.4 [27.5–40.2]^a^	26.0 [23.7–33.0]^a^	35.4 [29.0–42.0]^a^
Rate Your Plate	55 [50–60]	57 [52–63]	56 [50–61]	58 [53–64]
Comorbidities				
HTN	13 (52 %)^a^	20 (8O%)^a^	6 (43 %)^a^	15 (83 %)^a^
DM2	1 (4 %)	6 (24 %)	1 (7 %)	4 (22 %)
Tobacco Use	5 (20 %)	7 (28 %)	5 (36 %)	6 (33)%
Disease Characteristics				
SELENA-SLEDAI	8 [2–10]	6 [3–10.5]	4 [0–3.5]	7 [3.5–11.25]
Disease duration, years	6 [2–10]	6 [3–11]	5.5 [l.8–9.3]	6 [2.8–10.5]
ACR Criteria, total number	6 [5–7]	5 [5–7]	5 [4–7]	5.5 [5–7]
Lupus Nephritis	18 (72 %)	17 (68 %)	10 (71 %)	14 (78 %)
Medication				
Prednisone				
0 mg/day	7 (28 %)	11 (44 %)	5 (36 %)	7 (39 %)
≤7.5 mg/day	8 (32 %)	4 (16 %)	5 (36 %)	2 (11 %)
>7.5 mg/day	10 (40 %)	10 (40 %)	4 (29 %)	9 (50 %)
Hydroxychloroquine	22 (88 %)	22 (88 %)	12 (86 %)	16 (89 %)
Other Immunosuppressants				
Cyclophosphamide	0 (0 %)	2 (8 %)	0 (0 %)	1 (6 %)
Mycophenolate	11 (44 %)	11 (44 %)	8 (57 %)	7 (39 %)
Azathioprine	4 (16 %)	1 (4 %)	2 (14 %)	0 (0 %)
Misc. Medications				
Statin	5 (20 %)	8 (32 %)	2 (14 %)	7 (39 %)
ACEI/ARB	16 (64 %)	15 (60 %)	9 (64 %)	12 (67 %)

Characteristics of the fish oil treatment group and olive oil placebo group, for both the intial randomized patient groups (*N*=50) and the completers (*N*=32). Data presented as number of patients (%) or median and interquartile range [IQ.R]. Statistically significant differences (*p*<0.05) comparing fish oil and placebo groups are indicated with a superscript a(^a^)

At study completion, 18 patients remained in the fish oil group and 14 in the placebo group. Six of the 18 dropouts were lost to follow-up, 3 in each arm representing 12 % of the initial study sample. These patients either self-reported by telephone or an immediate family member confirmed they had continued taking the study medication without side-effects, but for various reasons failed to attend their study completion appointment. Additional reasons for dropout included requirement to take what was considered too many pills by patients including 2 in the fish oil group and 3 in the placebo group (*p* > 0.99). Side-effects that were cited as the cause for dropout were all gastrointestinal (2 fish oil and 5 placebo *p* = 0.42) and not significant (burping, 1 in each arm *p* > 0.99; nausea, 3 in the placebo group, *p* = 0.23; diarrhea, 1 in the fish oil group, *p* > 0.99; and abdominal pain, 1 in the placebo group, *p* > 0.99). Additionally, two completers reported the side-effect of burping (1 in each arm). Completers reported the ease of treatment as being very difficult (*n* = 2, 6 %), somewhat difficult (*n* = 15, 47 %), neutral (*n* = 9, 28 %), somewhat easy (*n* = 3, 9 %), and easy (*n* = 3, 9 %), with no significant difference related to the treatment group (*p* = 0.897). All completers consumed greater than 50 % of their treatment. Fifteen of eighteen (83 %) fish oil subjects and nine of fourteen (64 %) placebo subjects consumed greater than 75 % of their treatment. When completers were asked if they thought they would purchase over-the-counter fish oil supplements and begin taking them regularly, 26 out of 32 (81 %) responded in the affirmative. To examine if the patients were blinded to treatment allocation, completers were asked if they believed they knew their treatment group. Eleven patients stated that they did not believe they knew whether they had received fish oil or olive oil placebo (6 fish oil and 5 placebo). Twelve (67 %) of fish oil and 9 (64 %) of placebo patients believed they were taking fish oil (*p* > 0.99). No patients believed they were receiving placebo. Medications at baseline and completion are shown in Table 2. The unblinded evaluator did not alter medications for the duration of the study. For accuracy, change in prednisone dose was analyzed as a continuous variable to determine if significant changes in dose occurred between groups, with no significant difference being found (*p* = 0.929). Hydroxychloroquine use was surprisingly near complete in the patients. Other immunosuppressant use did not vary significantly between groups. Statin use was similar at baseline between groups (*p* = 0.235), but significantly higher in the fish oil patients at completion (*p* = 0.0276). McNemar’s test did not note a significant difference between baseline and completion of statin use for the fish oil group (*p* = 0.248) or the placebo group (*p* > 0.999).

**Table 2:** Medications at baseline and six months

	Placebo (*N*=14)		Fish oil (*N*=18)	
	Baseline	Six months	Baseline	Six months
Prednisone				
0 mg/day	5 (36 %)	7 (50 %)	7 (39 %)	5 (28 %)
≤7.5 mg/day	5 (36 %)	4 (29 %)	2 (11 %)	1 (6 %)
>7.5 mg/day	4 (29 %)	3 (21 %)	9 (50 %)	12 (67 %)
Median [IQR] mg/day	5 [0–20]	3 [0–9]	8 [0–20]	10 [0–15]
Hydroxychloroquine	12 (86 %)	14 (100 %)	16 (89 %)	16 (89 %)
Other Immunosuppressants				
Cyclophosphamide	0 (0 %)	1 (7 %)	1 (5 %)	1 (5 %)
Mycophenolate	8 (57 %)	7 (50 %)	7 (39 %)	10 (56 %)
Azathioprine	2 (14 %)	2 (14 %)	0 (0 %)	0 (0 %)
Misc. Medications				
Statin	2 (14 %)	2 (14 %)	7 (39 %)	10 (56 %)
ACE/ARB	9 (64 %)	9 (64 %)	12 (67 %)	14 (78 %)

Background medications for all completers at baseline and study completion. Data presented as number of patients (%) or median and interquartile range [IQR]

### Efficacy of fish oil quality of life/fatigue

**Fig. 2:**
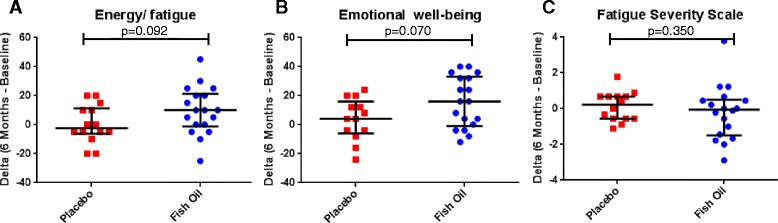
Quality of Life Assessments. RAND Short Form-36 (SF-36) Dot plot of score changes from baseline to six months for **a** Energy/fatigue (*p* = 0.092) and **b** Emotional Well- Being (*p* = 0.070), a higher score is indicative of better quality of life or fatigue; therefore a positive delta denotes improvement. Fatigue Severity Scale **c** Dot plot of score changes from baseline to six months (*p* = 0.350), a lower score indicates less fatigue; therefore a negative delta denotes improvement. For all graphs, center bar represents median and upper and lower bars are the 25^th^ and 75^th^ percentiles. Data were analyzed with Mann–Whitney U tests

Comparison of score changes for the Energy/fatigue subscale of the Rand SF-36 utilizing the Mann–Whitney test yielded a trend in improvement for the fish oil group (median 10.00 [IQR - 1.25 – 21.25]) compared to the placebo group (−2.50 [−6.25 – 11.25], *p* = 0.092; Fig. 2a).

Another related subscale, Emotional well-being also demonstrated a trend in improvement in the fish oil group (16.00 [−1.00 – 33.00] compared to the placebo group (4.00 [−5.00 – 14.00], *p* = 0.070; Fig. 2b). The FSS change scores were quite similar between the fish oil patients (− 0.056 [−1.500 – 0.500] and placebo patients (0.222 [−0.556–0.667], *p* = 0.350; Fig. 2c).

**Fig. 3:**
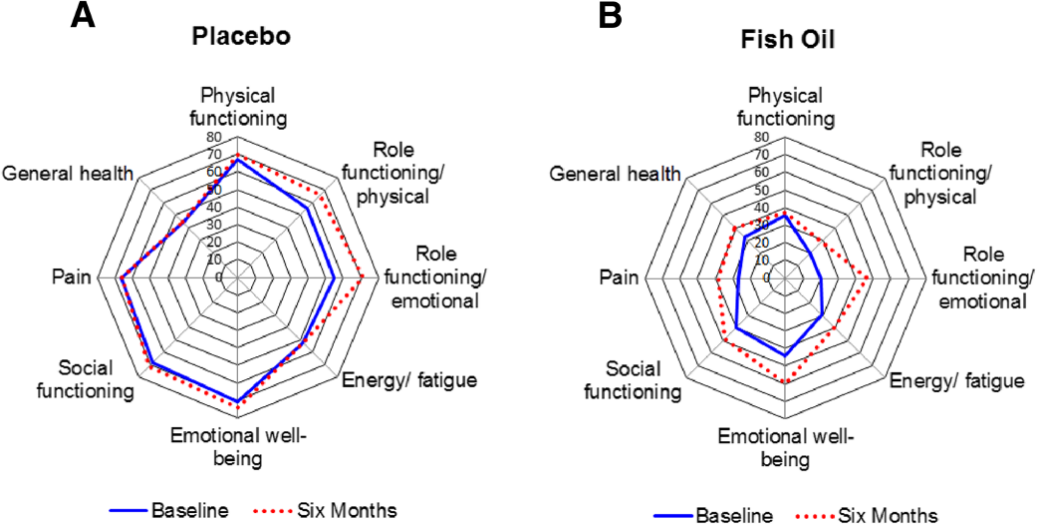
Quality of Life Assessments. RAND Short Form-36 (SF-36) Radial representation of the means of the 8 subscales as assessed at baseline (solid line) and six months (dotted line). **a** Placebo completers (*N* = 14) & **b** Fish Oil completers (*N* = 18)

Inexplicably, the fish oil group reported lower scores at baseline for the RAND SF-36 and higher scores on the FSS, indicating worse quality of life and worse fatigue (Table 3). The radial diagrams representing all 8 subscales of the RAND SF-36 allow better visualization of the baseline differences as well as areas with greater improvement (Fig. 3).

**Table 3:** RAND SF-36 at baseline, six months, and baseline-six months

	Placebo (*N*=14)	Fish oil (*N*=18}	Mann–Witney U
	Median [IQR]	Median [IQR]	*p*-value
Baseline			
Physical functioning	75.00 [52.90–90.00]	32.5 [17.50–55.00]	.004
Role functioning/physical	75.00 [0.00–100.00]	0.00 [0.00–25.00]	.022
Role functioning/emotional	66.67 [0.00–100.00]	0.00 [0.00–16.67]	.021
Energy/fatigue	55.00 [28.75–76.25]	20.00 [10.00–46.25]	.016
Emotional well-being	68.00 [60.00–89.00]	40.00 [28.00–64.00]	.006
Social functioning	68.75 [46.88–90.63]	37.50 [21.88–62.50]	.006
Pain	62.50 [45.00–92.50]	22.50 [9.38–47.50]	.001
General health	42.50 [28.75–62.50]	30 [18.75–50.00]	.100
Six Months			
Physical functioning	77.50 [48.75–96.25]	30.00 [15.00–51.25]	.005
Role functioning/ physical	75.00 [43.75–100.00]	0.00 [0.00–75.00]	.015
Role functioning/ emotional	100.00 [50.00–100.00]	16.67 [0.00–100.00]	.217
Energy/fatigue	55.00 [32.50–70.00]	37.50 [20.00–56.25]	.138
Emotional well-being	78.00 [61.00–88.00]	66.00 [44.00–77.00]	.037
Social functioning	68.75 [50.00–100.00]	50.00 [25.00–75.00]	.023
Pain	72.50 [46.25–92.50]	32.5 [22.50–57.50]	.009
General health	52.50 [20.00–60.00]	37.50 [30.00–51.25]	.380
Six Months-Baseline			
Physical functioning	5.00 [-10.00–21.25]	-2.50 [-10.00–16.25]	.468
Role functioning/physical	0.00 [0.00–25.00]	0.00 [0.00–25.00]	.878
Role functioning/emotional	0.00 [0.00–33.33]	0.00 [0.00–50.00]	.693
Energy/fatigue	-2.50 [-6.25–11.25]	10.00 [-1.25–21.25]	.092
Emotional well-being	4.00 [-5.00–14.00]	16.00 [-1.00–33.00]	.070
Social functioning	6.25 [-9.38–18.75]	6.25 [-12.50–37.50]	.657
Pain	-11.25 [-14.38–18.13]	10.00 [0.00–25.00]	.105
General health	-2.50 [-10.00–6.25]	5.00 [-11.25–23.75]	.312

RAND SF-36 data are presented as median and interquartile range (IQR) for all completer patients at baseline, study completion, and difference between baseline and six months

### Disease activity

**Fig. 4:**
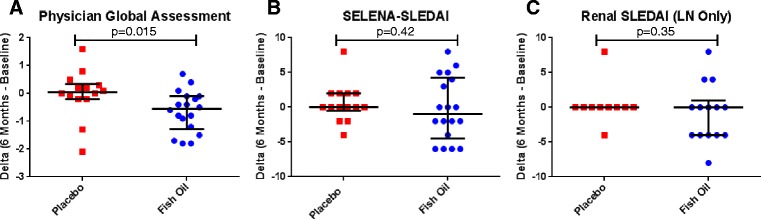
Disease Activity Assessments. Physician’s Global Assessment **a** Dot plot of score changes (*p* = 0.015), a higher score is indicative of worse overall disease activity; therefore a negative delta denotes improvement. SELENA-SLEDAI **b** Dot plot of change scores (*p* = 0.42) and **c** Renal component delta for LN patients only, Placebo *N* = 10 and Fish Oil *N* = 14, (*p* = 0.35), a higher score is indicative of higher disease activity; therefore a negative delta denotes improvement. For all graphs, center bar represents median and upper and lower bars are the 25^th^ and 75^th^ percentiles. Data were analyzed with Mann–Whitney U tests

In addition to evaluating fatigue, we assessed disease activity using the PGA and SELENA-SLEDAI. The fish oil patients exhibited improvement in global disease activity (−0.550 [−1.275– - 0.100] compared to the placebo patients (0.50 [−0.200–0.350]) based on the PGA (*p* = 0.015; Fig. 4a). The change in SELENA-SLEDAI (fish oil −1.00 [−4.5–4.25] and placebo 0.00 [− 0.50–2.00]) and the change in renal SELENA-SLEDAI (24 lupus nephritis patients only; fish oil 0.00 [−4.00–1.00] and placebo 0.00 [0.00–0.00]) scores did not indicate a significant difference between the two groups (*p* = 0.417 and *p* = 0.350, respectively; Fig. 4b and c). Baseline comparison of disease activity measures in the trial completers did not indicate significant difference between the groups.

### Changes in bomarkers

**Fig. 5:**
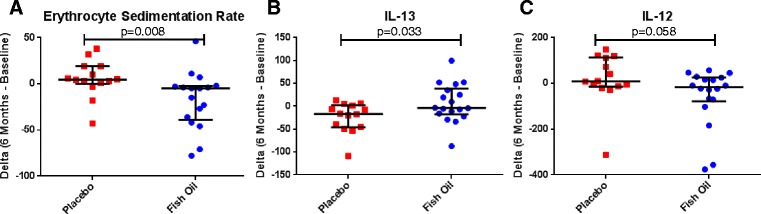
Biomarkers. **a** Erythrocyte Sedimentation Rate (ESR) (*p* = 0.008), reduced in fish oil group **b** IL-13 (*p* = 0.033) elevated in fish oil group **c** IL-12 (*p* = 0.058), reduced in fish oil group. For all graphs, center bar represents median and upper and lower bars are the 25^th^ and 75^th^ percentiles. Data were analyzed with Mann–Whitney U tests

Additional laboratory tests were evaluated for changes during treatment. ESR, an accepted measure of systemic inflammation, demonstrated a significant reduction in the fish oil group compared to the placebo group (fish oil −5.0 [−39.0– -2.5] and placebo 4.5 [0.0–19.0], *p* = 0.008; Fig. 5a). Among the cytokines/chemokines/growth factors studied, we observed an increase in the level of IL-13 (fish oil −3.89 [−17.37–38.55] and placebo −16.86 [−46.25–2.31], *p* = 0.033; Fig. 5b) and a decrease in the level of IL-12 (fish oil −16.13 [−78.50–26.37] and placebo 8.54 [−14.16–113.35], *p* = 0.058); Fig. 5c).

## Discussion

Fatigue and reduced quality of life commonly impact SLE patients. Metabolomic profiling in SLE alluded to a reduction in markers of energy generation as well as increased markers of inflammation [4]. These alterations may manifest physically and mentally as ill-defined symptoms of fatigue, generalized pain, and depressed mood. Also noted in the metabolomic study was a reduction in serum omega-3 fatty acids [4].

Supplementation with omega-3 fatty acids through fish oil has resulted in improvement in quality of life in diseases other than lupus. This trial is the first to investigate the impact of fish oil supplementation on fatigue and quality of life in patients with SLE. We have also evaluated disease activity and a variety of serum biomarkers. We found that fish oil supplementation resulted in improvement in quality of life, disease activity, and biomarkers of inflammation.

The Fatigue Severity Scale did not appear to be a metric capable of capturing subtle changes in a small group of patients, as majority of scores remained approximately the same at both time points. However, the RAND SF-36 did indicate positive improvement trends for the Energy/fatigue and Emotional well-being subscales. Due to the large difference in baseline values the results are somewhat difficult to interpret. Attempt to incorporate baseline values as a covariate resulted in loss of improvement signals in this small group of patients. Our results are consistent with fish oil studies in other disorders. A meta-analysis found fish oil to have a positive effect on major depressive disorder, using various validated depression metrics [27]. Included in that meta-analysis is a study of elderly depressed women that included SF-36 evaluation, where they noted improvement in the mental health (equivalent to Emotional well-being) subscale, though no clear difference was seen in the vitality (Energy/fatigue) subscale [17]. Fish oil supplementation improved quality of life in patients with dry eyes [28]. Evaluation of 3 clinical trials in multiple sclerosis concluded fish oil to be ineffective for fatigue and quality of life [29]. Our results suggest that fish oil may be beneficial for particular aspects of health related quality of life and further studies in this area are warranted.

Disease activity was assessed using the SELENA-SLEDAI and PGA. There was not a clear difference in SELENA-SLEDAI between the groups. However, the PGA indicated a positive signal with fish oil therapy. Perhaps the PGA integrated aspects of mental health and quality of life not captured by the SLEDAI as well as contextualizing aspects of disease activity that may be more or less relevant in a particular patient. Fish oil has been noted to have variable effects on disease activity in previous studies [7, 8, 11–14]. The amount and duration of the fish oil used varied widely in those prior studies. In a meta-analysis of several chronic renal diseases including IgA nephropathy, diabetic nephropathy, and one study of lupus nephritis, fish oil was found to have the beneficial effect of reducing proteinuria, but not improving GFR [19]. Reduced proteinuria, improved GFR, and improved survival were treatment effects of fish oil in murine lupus nephritis models [30–33]. The majority of our patients had lupus nephritis (LN). Hence this subset was evaluated for renal-specific parameters of the SLEDAI as well as their spot protein to creatinine ratios, though fish oil did not appear to strongly impact either. A previous clinical trial found similar results [12].

Multiple biomarkers were assessed including markers of inflammation, cytokines, chemokines, and growth factors. ESR, assessed through standard of care laboratory testing, was found to be significantly reduced in the fish oil group as compared to the placebo. This finding is an indication of a reduction in inflammation in these patients. Prior studies did not report a change in ESR in SLE patients receiving fish oil compared to placebo [8, 11]. However, ESR was found to be reduced in peritoneal dialysis patients [34] and rheumatoid arthritis patients given fish oil [35]. The role of IL-13, a T-cell secreted anti-inflammatory cytokine, in SLE and LN is quite complex as active LN patients have elevated serum levels and increased kidney tissue transcription of the *IL13* gene [36]. We found that treatment with fish oil increased the level of IL-13 as compared to placebo. Cultured lymph node cells from a mouse model of allergic airway disease also noted increased IL-13 in cells derived from the fish oil fed mice [37]. Fish oil supplementation also increased IL-13 in lung tissue of a murine model of allergic inflammation [38]. It is conceivable that the increased Th2 skewing induced by the elevated IL- 13 may be beneficial in SLE. IL-12 is a pro-inflammatory cytokine that is elevated in SLE [36]. We find fish oil supplementation reduces serum IL-12 in SLE patients as compared to placebo. In a mouse model of SLE, MRL/*lpr*, fish oil supplementation was found to reduce IL-12 serum levels. Infectious challenge resulted in reduced IL-12 level in fish oil fed healthy mice [39, 40]. Omega-3 fatty acids have been noted to have anti-inflammatory properties including alteration to cytokine signaling and anti-oxidant effects in a recent mechanistic study [10]. Our findings of fish oil supplementation resulting in reduced ESR and IL-12, as well as increased IL-13 are in concordance with published human and animal studies. Importantly, these results add to the plausibility of fish oil resulting in reduced inflammation through multiple molecular mechanisms, including alterations in Th1/Th2 balance.

We recognize that our study had several limitations. This study was underpowered for the fatigue and quality of life outcomes. Additionally, although randomization resulted in two similar groups based on demographics with the exception of older age and higher BMI in the fish oil group, the latter group had worse quality of life and fatigue at baseline. Age and BMI may have contributed to the disparity in baseline quality of life measures. Randomization is performed in order to match groups on both measured variables as well as unmeasured variables. In large studies randomization results in well matched groups, however in smaller studies random differences in measured variables are still possible. The major cause of dropout was failure to follow-up in clinic. Studies with dropouts less than 20-30 % are acceptable for performing unmodified intention-to-treat analysis and imputation, however our study resulted in 36 % dropouts [41, 42]. Although the patients were blinded to treatment, the investigator was able to discern the treatment groups. Besides physician global assessment and selected components of the SLEDAI, all remaining measures were objective or patient-reported. A portion of fish oil patients believed they were un-blinded to their treatment group; however a similar number of placebo patients incorrectly believed they were taking fish oil. Multiple patients were undergoing therapy with immunosuppressives and glucocorticoids, which was unavoidable as we desired the inclusion of patients with active disease. Future trial design could benefit from a placebo run-in period to minimize dropout, inclusion of larger numbers of patients, stratification to ensure balanced treatment groups, double-blinding, and unmodified intention-to-treat analysis.

## Conclusions

This study indicates fish oil has potential benefits in SLE. Fish oil is a minimal risk, widely available oral supplement. The strengths of this study were the inclusion of a concurrent placebo arm, randomization of the two groups, and patients being blinded to their treatment. An additional strength is the novel assessment of fish oil’s effect on quality of life, fatigue, and a large panel of soluble mediators in SLE patients. Although the study evaluated a small group of patients, there were positive indications in the treatment group for quality of life, fatigue, disease activity, and inflammation biomarkers. Further studies are warranted to confirm our promising findings.

## Abbreviations

ACEI/ARB: Angiotensin Converting Enzyme-Inhibitor/Angiotensin Receptor Blocker
ACR: American College of Rheumatology
ANOVA: Analysis of Variance
BMI: Body mass index
C3: Complement 3
C4: Complement 4
CCL: C-C motif chemokine ligand
CRP: C- reactive protein
CXCL: C-X-C motif chemokine ligand
DHA: Docosahexaenoic acid
DM2: Type 2 Diabetes mellitus
dsDNA: Double-stranded DNA
EGF: Epidermal growth factor
EPA: Eicosapentaenoic acid
ESR: Erythrocyte sedimentation rate
FGF: Fibroblast growth factor
FSS: Fatigue Severity Scale
G- CSF: Granulocyte-colony stimulating factor
GM-CSF: Granulocyte-macrophage colony- stimulating factor
HGF: Hepatocyte growth factor
HMG-CoA: 3-Hydroxy-3-methyl-glutaryl- coenzyme A
HTN: Hypertension
IFN: Interferon
IL: Interleukin
IP-10: Interferon gamma-induced protein 10
LDL: Low-density lipoprotein
LN: Lupus nephritis
MCP: Monocyte chemotactic protein
MDA: Malondialdehyde
MIG: Monokine induced by gamma interferon
MIP: Macrophage inflammatory protein
PGA: Physician Global Assessment
RAND SF-36: RAND 36-Item Short Form Health Survey Version 1.0
RANTES: Regulated on activation, normal T cell expressed and secreted
RYP: Rate Your Plate
SELENA-SLEDAI: Safety of Estrogens in Lupus Erythematosus National Assessment – Systemic Lupus Erythematosus Disease Activity Index
sICAM-1: Soluble intercellular adhesion molecule 1
SLE: Systemic lupus erythematosus
sVCAM-1: Soluble vascular cell adhesion molecule 1
TBARS: Thiobarbituric Acid Reactive Substances
Th: T helper cell
TNF: Tumor necrosis factor
VEGF: Vascular endothelial growth factor
VLDL: Very low-density lipoprotein
